# Electroencephalograph-Based Emotion Recognition Using Brain Connectivity Feature and Domain Adaptive Residual Convolution Model

**DOI:** 10.3389/fnins.2022.878146

**Published:** 2022-06-22

**Authors:** Jingxia Chen, Chongdan Min, Changhao Wang, Zhezhe Tang, Yang Liu, Xiuwen Hu

**Affiliations:** School of Electronic Information and Artificial Intelligence, Shaanxi University of Science and Technology, Xi’an, China

**Keywords:** EEG, brain connectivity, residual convolution, domain adaptative, emotion recognition

## Abstract

In electroencephalograph (EEG) emotion recognition research, obtaining high-level emotional features with more discriminative information has become the key to improving the classification performance. This study proposes a new end-to-end emotion recognition method based on brain connectivity (BC) features and domain adaptive residual convolutional network (short for BC-DA-RCNN), which could effectively extract the spatial connectivity information related to the emotional state of the human brain and introduce domain adaptation to achieve accurate emotion recognition within and across the subject’s EEG signals. The BC information is represented by the global brain network connectivity matrix. The DA-RCNN is used to extract high-level emotional features between different dimensions of EEG signals, reduce the domain offset between different subjects, and strengthen the common features between different subjects. The experimental results on the large public DEAP data set show that the accuracy of the subject-dependent and subject-independent binary emotion classification in valence reaches 95.15 and 88.28%, respectively, which outperforms all the benchmark methods. The proposed method is proven to have lower complexity, better generalization ability, and domain robustness that help to lay a solid foundation for the development of high-performance affective brain-computer interface applications.

## Introduction

Emotion is one of the most significant perceptual factors that affect our personal and social behavior. Recognizing the user’s emotional state can better enhance the user’s experience in human-computer interactions. Emotion recognition aims to detect and model human emotions in the process of human-computer interactions. At present, the signals used to represent human emotional states include facial expressions, voice signals, and some physiological signals (Calvo and D’Mello, 2010). An electroencephalograph (EEG) is a kind of effective biological signal which could reflect a human emotional state with high time resolution. It has obvious advantages such as not being easy to disguise, being more objective, and containing more comprehensive context information (Peng et al., 2021). At present, EEG-based emotion recognition has become an important research topic in the field of emotion computing.

Recently, different EEG emotion recognition methods have been proposed. Peng et al. (2021) proposed a graph regularized least squares regression with feature importance learning (GFIL) for joint feature importance learning and emotion recognition. Mert and Akan (2018) used the time-frequency distribution of the EEG signal obtained by the multiple synchronous compression transformation to classify the valence and arousal emotion. To better identify the potential complex features in EEG signals, researchers turned their attention to deep learning models to explore deep emotion-related features. Chen et al. (2019b) proposed an emotional feature learning and classification method based on time-frequency feature fusion and a deep convolutional neural network, whose performance is 3.58 and 3.29% higher than that of the best traditional BT classifier in valence and arousal, respectively. The author of this article also proposed a hierarchical two-way gated recurrent unit (GRU) network (Chen et al., 2019a), which introduces an attention mechanism in the time point and time segment levels to learn more discriminative feature expression through highlighting the contributions of important time points and segments to emotion prediction. Zheng and Lu (2015) used differential entropy (DE) features as the input of the deep belief network (DBN) for three-category emotion recognition. Li et al. (2018) input the 2D topographic map of DE features in the spatial arrangement of EEG electrodes into CNN to make positive, neutral, and negative emotion classifications.

However, in the practical application of brain-computer interface (BCI), the distribution of EEG data varies greatly among different individuals and different tasks, so the model trained on one subject’s or on one task’s EEG data is hardly used to accurately predict the emotional types of other subjects or other tasks. Xin et al. (2017) proposed an adaptive subspace feature matching (ASFM) strategy to make three-type emotion classification on the SEED data set and achieved average accuracy and SD of 80.46 and 6.48%. Li R. et al. (2021) proposed a multi-domain adaptive graph convolutional network (MD-AGCN) to effectively extract complementary domain information and channel relationships for EEG-based emotion recognition. Wang et al. (2020) proposed a cross-subject emotion recognition method based on a convolutional neural network and depth domain confusion (DDC) algorithm to reduce the feature distribution difference between the source domain and the target domain and obtained an average accuracy of 82.16% and a standard deviation of 4.43% for the cross-subject experiment on SEED data set. Li et al. (2022) propose a novel method for EEG-based emotion recognition, which can learn multiple tasks simultaneously while exploiting commonalities and differences across tasks and characterize the intrinsic relationship among various EEG channels according to their weight learned by the attention capsule network (CapsNet). Li C. et al. (2021) also proposed a novel neural architecture search (NAS) framework based on reinforcement learning (RL) for EEG-based emotion recognition and the experimental results demonstrated that their proposed NAS outperforms the state-of-the-art CNN-based methods.

Recently, research (Costa et al., 2006; Lee and Hsieh, 2014) on brain science confirmed that there is unique gain information related to brain cognition between different brain regions, including emotional tendencies. Murias et al. (2007) found that there is a strong EEG connection among patients with autism spectrum disorder at rest. Whitton et al. (2018) believe that the increase in high frequency bands based on the functional connection of EEG signals may be a neural model for the recurrence of major depression. Toll et al. (2020) identified brain regions whose frequency-specific, orthogonalized resting-state EEG power envelope connectivity differs between combat veterans with posttraumatic stress disorder (PTSD) and healthy combat-exposed veterans, and determined the behavioral correlates of connectomic differences. Zhang et al. (2021) reported the identification of two clinically relevant subtypes of PTSD and major depressive disorder (MDD) based on robust and distinct brain functional connectivity patterns from high-density resting-state EEG. Yi et al. (2021) constructed the EEG large-scale cortical dynamical functional network connectivity (dFNC) based on a brain atlas to probe the subtle dynamic activities in the brain and developed a novel wavelet coherence-S estimator (WTCS) method to assess the dynamic couplings among functional subnetworks with different spatial dimensions and demonstrated the robustness and availability of dFNC through a simulation study. Therefore, it is of great significance to explore the features of functional brain networks based on EEG signals. Wu et al. (2019) developed a key sub-network method that uses topological features to identify emotional states based on EEG signals. Zhong et al. (2020) proposed a regularized graph neural network that uses frequency domain information from EEG data and pre-calculated frequency features from EEG signals to study the relationship between channels for emotion recognition. Mauss and Robinson (2009) proposed that emotional processes should be considered to involve spatial distribution features, rather than limited to isolated specific brain regions. Although the research on EEG-based emotion recognition has made great progress, it is still difficult to obtain emotion-related brain network features from the original EEG signal because the EEG signal itself is relatively weak and easily interfered with by noise. An increasing number of deep learning methods are applied to emotion recognition. With the continuous improvement of the depth and complexity of the model, there are higher requirements for the computing power of the servers. These problems such as high model complexity, gradient explosion, overfitting, and so on, under the premise of ensuring high prediction performance, need to be solved urgently.

To this end, this study proposes an end-to-end emotion recognition method based on brain connectivity (BC) features and domain adaptation residual convolution (BC-DA-RCNN). At first, we rearrange the order of all electrodes according to specific brain network standards and use three connectivity measurement algorithms to calculate the connectivity value between each channel, in which the electrode position is taken as the node, and the connectivity value is taken as the weight between the nodes to construct the feature matrix for integrating global brain network information. Then, we build a residual convolution model and introduce the domain discriminant loss to constrain the invariant feature space, reduce data differences between subjects and learn the consistent and deep features cross subjects. At last, we carry out a lot of comparative experiments on the DEAP data set to verify the effectiveness of the proposed method. The rest of this article is arranged as follows: In section “Materials and Methods,” we describe the overall framework of the proposed method in detail. In section “Experiments and Results,” we make experimental verification, display, and analyze the experimental results. In section “Discussion,” we discuss and analyze the key innovations and limitations of the proposed method. Finally, the conclusion is presented.

## Materials and Methods

### Construction of Connectivity Feature Matrix

**FIGURE 1:**
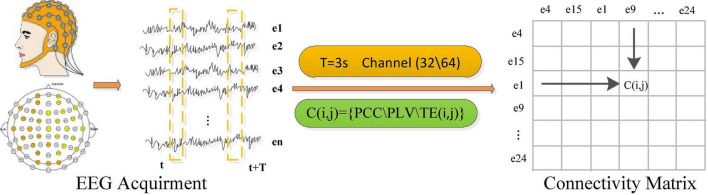
EEG signal acquisition and connectivity feature representation.

Electroencephalograph-based emotional brain-computer interface systems usually use portable and wearable multi-channel electrode caps to collect signals. When the subject watched the stimulation video, the sensor on the electrode cap can capture the fluctuation of the subjects’ brain scalp current. The overall process of EEG signal acquisition and feature conversion is shown in Figure 1, which shows the electrode position distribution on a commonly used BCI electrode cap. The number and distribution of the electrodes or channels in different BCI systems are also different. The sensor readings obtained by the EEG acquisition system represent the time series of EEG signals at a certain sampling frequency. Usually, the EEG sequences collected are expressed as $S_{n}={\left\{s_{n}^{1},s_{n}^{2},\ldots ,s_{n}^{T}\right\}}^{N\times f_{s}}$, where *T* represents the length of the time series (the length of window), *n* represents the electrode no., *N* represents the total number of electrodes, and *f*_*s*_ represents the sampling frequency, $s_{n}^{t}$ represents the reading value of the *n*th electrode at time point t. Based on the EEG sequences of all adjacent channels, different connectivity measurement algorithms are used to calculate the connectivity characteristic value between channels, thereby constructing an EEG connectivity feature matrix.

Four methods including Pearson correlation coefficient (PCC) (Benesty et al., 2009), phase lock value (PLV) (Lachaux et al., 1999), transfer entropy (TE) (Schreiber, 2000), and wavelet coherence coefficient (WCC) (Grinsted et al., 2004) are selected to calculate the connectivity feature of the original EEG signals between different electrodes in the cerebral cortex, therefore four types of connectivity feature matrices in time series dimension are constructed, respectively. The connectivity features of all electrode pairs are represented as a matrix, where element C (*i*, *j*) represents the connectivity between the EEG signals obtained from the *i*-th and *j*-th electrodes, as shown in the matrix on the right side of Figure 1. This connectivity matrix is equivalent to an adjacency matrix to a graph, where EEG electrodes are considered nodes and connectivity values are considered edge weights. The arrangement and driving mode of electrodes in the connectivity matrix is very important for the evaluation of brain network connectivity. EEG data extracted from different brain regions contain a lot of spatial information. Thus, it is very important to use the relationship between the electrodes to obtain the emotion-related connectivity gain information. Literature (van den Broek et al., 1998) found that the electrode position when extracting EEG is determined according to the conductance effect of the human body, and the EEG obtained from adjacent brain regions tends to be similar.

**FIGURE 2:**
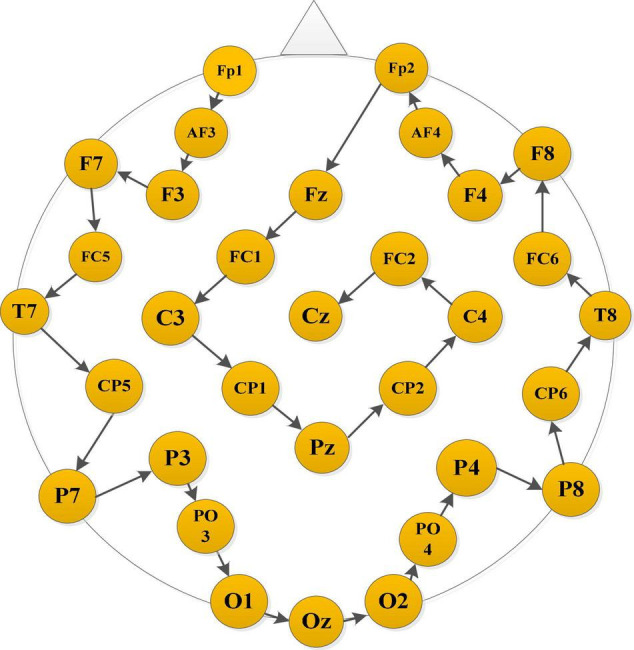
Brain data-driven approach.

In order to construct a smoothly connected matrix carrying spatial information, we rearrange the order of electrodes based on their distance. Specifically, starting from the electrode in the left frontal area, the one closest to the current electrode is selected as the next electrode. The rearranged result of the 32 electrodes in the DEAP data set is Fp1→AF3→F3→F7→FC5→T7→CP5→P7→P3→PO3→O1 →O_Z_→O2→PO4→P4→P8→CP6→T8→FC6→F8→F4→ AF4→Fp2→Fz→FC1→C3→CP1→Pz→CP2→C4→FC2→ Cz, as shown in Figure 2. Then, the following four connectivity measurement algorithms are used to calculate the connectivity value between adjacent channels of the rearranged EEG electrodes sequence:

#### Pearson Correlation Coefficient

The Pearson correlation coefficient can express the linear relationship between two signals as a continuous number from −1 to 1, where −1 and 1, respectively, indicate a perfect positive and negative linear relationship, and 0 indicates that two signals are uncorrelated. The closer the correlation coefficient is to 1 or −1, the stronger the correlation is. The closer the correlation coefficient is to 0, the weaker the correlation is. Let $S_{i}=\left\{s_{i}^{1},s_{i}^{2},\ldots ,s_{i}^{T}\right\}$ denote the EEG sequence of the *i*-th electrode, where *T* denotes the time length of the signal. The value of the PCC feature matrix for EEG between the *i*-th and *j*-th channel is calculated as follows:


(1) $$P\,C\,C\,\left(i,j\right)=\frac{\frac{1}{T}\,\sum_{t=1}^{T}\left(s_{i}^{t}-\mu_{i}\right)\,\left(s_{j}^{t}-\mu_{j}\right)}{\sigma_{i}∙\sigma_{j}}$$

where, *μ*_*i*_ and *σ*_*i*_ represent the mean and variance of the EEG sequence on the *i*-th channel, *μ*_*j*_ and *σ*_*j*_ represent the mean and variance of the EEG sequence on the *j*-th channel, $s_{i}^{t}$ and $s_{j}^{t}$, respectively, represent the EEG readings on the *i*-th and *j*-th channels at time *t*, and Σ stands for the cumulative sum.

#### Phase Locking Value

Phase locking value is a statistic value used to estimate the instantaneous phase relationship between EEG of different electrodes and study the synchronous changes of task-induced brain neural activity. First, the 4–45 hz bandpass filtering is applied to the original EEG signals. The instantaneous phase value of the EEG sequence on each electrode is calculated by Hilbert transform, and then the PLV feature matrix is calculated by averaging their absolute phase difference as follows:


(2) $$P\,L\,V\,\left(i,j\right)=\frac{1}{T}\,\left|\sum_{t=1}^{T}\exp\left\{u\,\left(\varphi_{i}^{t}-\varphi_{j}^{t}\right)\right\}\right|$$

where *T* represents the time sequence length of the signal, ^ϕ*t*^ represents the instantaneous phase value of each electrode at time t calculated by Hilbert transform, $\phi_{i}^{t}-\phi_{j}^{t}$ represents the phase difference of the *i*-th and *j*-th electrode at time *t*, $\exp\{u\left(\varphi_{i}^{t}-\varphi_{j}^{t}\right)$ means to obtain the complex signal by Euler formula on the phase difference, and then the amplitudes of these complex signals at all-time points in an EEG sample window are accumulated and averaged as the value of PLV(*i*, *j*) which is between 0 to 1. The value of 1 means the two electrodes’ signals are completely synchronized, and 0 means the two electrodes’ signals are completely independent. The closer to 1 the PLV is, the smaller the phase difference. The closer to 0 the PLV is, the greater the phase difference. PLV can better estimate brain functional connectivity. If the PLV value on some electrodes rises or falls together, the synchrony among these electrodes is enhanced; otherwise, the synchrony among these electrodes decreases.

#### Transfer Entropy

The transfer entropy well describes the information flow between two directional signals *x*_*i*_ and *x*_*j*_. TE(i→j) is a special conditional mutual information, which takes ${\text{s}}_{\text{j}}^{\text{t}}$ as the conditional variable and then calculates the mutual information between ${\text{s}}_{\text{j}}^{\text{t}+1}$ and ${\text{s}}_{\text{i}}^{\text{t}}$ with the following formula:


(3) $$\text{TE}\left(\text{i}\to \text{j}\right)=\frac{1}{\text{T}-1}\sum_{\text{t}=1}^{\text{T}-1}\text{P}\left({\text{s}}_{\text{i}}^{\text{t}},{\text{s}}_{\text{j}}^{\text{t}},{\text{s}}_{\text{j}}^{\text{t}+1}\right)\cdot \text{log}\frac{\text{P}\,\left({\text{s}}_{\text{j}}^{\text{t}+1}|{\text{s}}_{\text{i}}^{\text{t}},{\text{s}}_{\text{j}}^{\text{t}}\right)}{\text{P}\,\left({\text{s}}_{\text{j}}^{\text{t}+1}|{\text{s}}_{\text{j}}^{\text{t}}\right)}$$

It also represents the dependence degree of the *j*-th channel’s EEG on the *i*-th channel’s EEG and the dynamic information shared between channel *i* and channel *j.*

#### Wavelet Coherence Coefficient

The wavelet coherence coefficient calculates the time-frequency coherence between two signals based on the wavelet transform, which can highlight the similarity of the local features. As EEG is a kind of non-stationary random signal, we use the wavelet coherence method to study the localized correlation in the time and frequency domain between EEG electrodes corresponding to different emotional states and reflect the time and frequency correlation intensity between electrodes in different brain regions. The wavelet coherence coefficient between different EEG electrodes is defined as:


(4) $$\text{W}\,C\,C=\frac{{\left|X\,\left(C_{i}^{{}^{*}}\,\left(a,b\right)\,C_{j}^{{}^{*}}\,\left(a,b\right)\right)\right|}^{2}}{X\,\left({\left|C_{i}\,\left(a,b\right)\right|}^{2}\right)\cdot X\,\left({\left|C_{j}\,\left(a,b\right)\right|}^{2}\right)}$$

Here, *C*_*i*_(*a*,*b*) and *C*_*j*_(*a*,*b*) represent the wavelet coefficients of the continuous wavelet transform of two EEG signals on the *j*-th electrode and the *i*-th electrode at the scale factor *a* and the translation factor *b*, respectively, the superscript * represents the complex conjugate, and *X* represents the smoothing operator of wavelet coefficients in time and scale. The WCC feature matrix is obtained by averaging the WCC matrices at all-time points of an EEG sample.

The above four connectivity measurement algorithms are used to calculate four types of BC feature matrices of each EEG sample as the matrix on the right side of Figure 1. These connectivity feature representations not only contain the inherent timing and spatial information of the original EEG data but also reflect the connectivity characteristics between different electrodes, which include more emotional spatial-temporal gain information and become the key to improving the performance of emotion recognition.

### Residual Convolutional Neural Network and Optimization

Deep neural networks have achieved a series of breakthroughs in image recognition. With the introduction of more complex neural networks, two important problems need to be solved: one is the increase in model complexity requires a large amount of computing power, and the other is the network performance degradation caused by hierarchical stacking and increased depth. We apply the residual structure in ResNet (He et al., 2016) to solve the problem of deep model performance degradation without increasing the complexity of the model and to make the network easier to optimize.

#### Residual Learning Module

**FIGURE 3:**
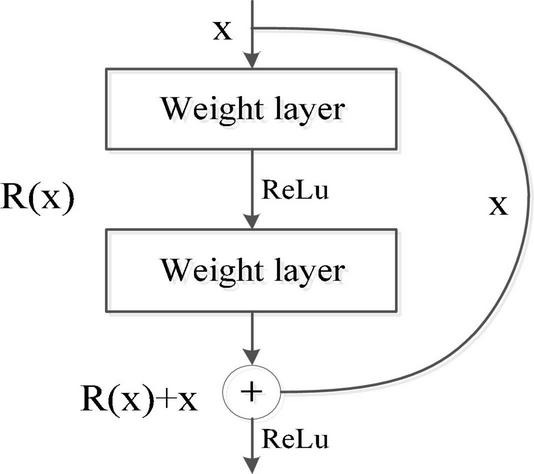
Residual learning process.

Inspired by ResNet, we introduce a residual learning module to optimize the convolutional neural network. Through the jump connection in residual learning, the gradient is transmitted to the lower layer, and the deeper features can be obtained in a relatively simple model. Therefore, we propose to use the residual convolutional neural network (RCNN) to replace the complex models to achieve better prediction results. The shortcut connection is the most prominent feature of ResNet. It only needs to perform identity mapping, without increasing additional parameters and computational complexity. The entire RCNN network is still trained end-to-end with a stochastic gradient descent (SGD) algorithm with back propagation. Specifically, the required underlying mapping is expressed as *H(x)*, where *x* represents the input of the first layer. If multiple nonlinear layers can approach a complex function asymptotically, so does the residual function, and the input and output dimensions in *H(x)-x* operation are the same. Let R(*x*) represent this approximate function as *R(x): = H(x)-x*. The original mapping *H(x)* is recast as *R(x)+x*, which can be realized by a feed forward neural network with shortcut connections, shown in Figure 3.

#### Residual Convolutional Neural Network

**FIGURE 4:**
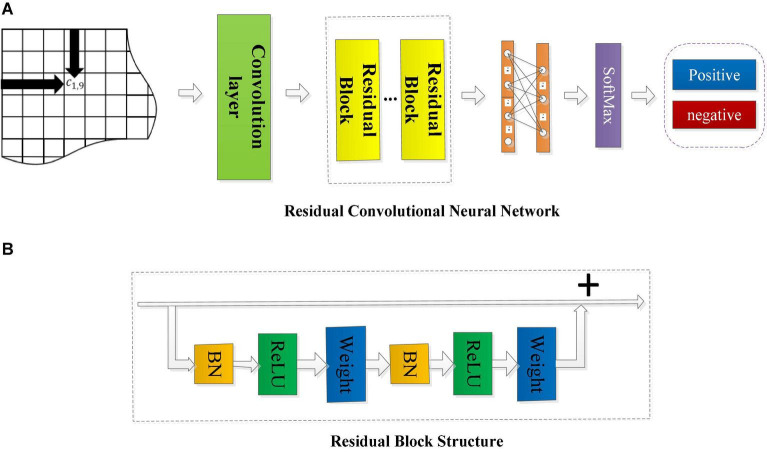
The framework of the residual convolutional neural network and the structure diagram of the residual block. **(A)** The framework of RCNN model. **(B)** The structure of residual block.

The overall structure of the RCNN is shown in Figure 4.

The input in Figure 4 is three types of connectivity feature matrices calculated by the above methods. In the first convolutional layer, different sizes of filters (3 × 3 and 5 × 5) are used to scan the input connectivity feature matrix to extract the spatial features of the EEG data. The learned features are then entered into the residual convolution module, in which a number of residual blocks and the kernel size will be discussed and analyzed later. After several residual blocks, the output is flattened and sent to a fully connected layer with a sigmoid activation function. Finally, the final feature vector is input to a SoftMax layer for emotion category prediction, and the output is the accuracy of emotion recognition.

Figure 4A shows that the residual convolutional neural network is composed of several residual layers, and the structure of each residual block is shown in Figure 4B. The shortcut connection is represented as a straight line in Figure 4B, and each residual mapping can be expressed as:


(5) $$y=F\,\left(x,\left\{W_{i}\right\}\right)+x$$

where *x* and *y* represent the input and output of the residual block, respectively, the function *F* = *W*_2_σ(*W*_1_⋅*x*) represents the residual mapping to be learned, σ is the ReLu activation function, and *W*_*1*_ and *W*_*2*_ represent two weight layers in the residual structure which has only two weight stack layers as shown in Figure 3. Here, *F* + *x* indicates that the residual structure is realized through shortcut connection and element-wise addition. Figure 4B shows that each residual block is composed of two layers of nonlinear transformations with a batch normalization (BN) layer and activation layer, respectively, to solve the inherent problem of performance degradation of the deep learning model.

In the residual convolution module, three different sizes of kernels (3 × 3, 5 × 5, 7 × 7) and 4 different numbers of residual blocks (*r* = 1, *r* = 3, *r* = 5, *r* = 7) are, respectively, used to construct the RCNN models to evaluate the impact of the kernel size and the number of residual blocks on the classification performance of the model. Dropout with a rate of 0.2 is used after the fully connected layer to avoid the model overfitting. The stochastic gradient descent method based on the Adam update rule is used to minimize the cross-entropy loss of the model. The learning rate is initialized to 0.0001. The proposed RCNN neural networks are all implemented with the TensorFlow framework and trained from scratch based on the NVIDIA Titan X Pascal GPU in a fully supervised manner. The detailed hyper parameters of the proposed RCNN model are shown in Table 4.

**TABLE 1:** EEG data and label format.

Scenarios	Feature type	Window length	EEG data shape	Label shape
Subject-dependent	PSD	3-s	32(channel) × 384(points) × 4600(epochs)	1 × 4,600
	PLV/PCC/TE/WCC	3-s	32(channel) × 32(channel) × 4600(epochs)	1 × 4,600
Subject-independent	PSD	3-s	32(channel) × 384(points) × 147200(epochs)	1 × 147,200
	PLV/PCC/TE/WCC	3-s	32(channel) × 32(channel) × 147200(epochs)	1 × 147,200

**TABLE 2:** Hyper parameters of the benchmark models on PLV feature.

Benchmark models	Input data size	Implementation details
SVM	[32 × 32, sample_size]	kernel = “rbf”, gamma = 8, *c* = 0.05
BT	[32 × 32, sample_size]	Method = bag, nLearn: 100, weak learner: Tree, Type: classification
CNN	[batch_size, feature_size]: [60, 32 × 32]	Hidden_layers = 2, hidden_size = 64, batch_size = 60, learning_rate = 0.005, dropout = 0.2, epochs = 120
LSTM	[batch_size, seq_len, channels]: [80, 32, 32]	Hidden_layers = 2, hidden_size = 64, batch_size = 120, learning_rate = 0.004, dropout = 0.2, epochs = 80, num_directions = 2
DBN	[batch_size, feature_size]: [60, 32 × 32]	Hidden_layers = 3, hidden_size = 64, batch_size = 60, learning_rate = 0.004, dropout = 0.2, epochs = 140
BiLSTM	[batch_size, seq_len, channels]: [60, 32, 32]	Hidden_layers = 2, hidden_size = 64, learning_rate = 0.008, dropout = 0.2, num_directions = 2, epochs = 200

**TABLE 3:** The classification results of the DA-RCNN model configured with different hyperparameters.

Blocks	Kernels_size	Arousal (%)			Valence (%)		
		Sp	Sn	Acc	Sp	Sn	Acc
1	3 × 3	69.54	80.70	75.12	72.42	79.10	75.76
	5 × 5	72.57	85.19	78.88	72.59	85.27	78.93
2	3 × 3	82.9	90.30	86.60	83.62	89.16	86.39
	5 × 5	80.36	93.70	87.03	81.07	93.25	87.16
3	3 × 3	88.24	97.66	92.95	89.12	97.38	93.25
	5 × 5	**90.78**	**98.90**	**94.84**	**91.88**	**98.42**	**95.15**
4	3 × 3	86.82	91.20	89.01	85.51	90.57	88.04
	5 × 5	85.53	90.55	88.04	85.65	91.81	88.73
5	3 × 3	82.88	88.47	85.73	82.78	89.06	85.92
	5 × 5	81.98	89.00	85.49	83.70	87.62	85.66

*Bold values represent the best comparative results.*

**TABLE 4:** The hyper parameters of the proposed BC-DA-RCNN model.

	Layer type	Size	Stride	Output shape
1	Input	32 × 32	–	None
2	Convolution layer	32 filters size of (3 × 3 or 5 × 5 or 7 × 7)	1	32 × 32 × 32
3	Residual block 1	32 filters size of (3 × 3 or 5 × 5 or 7 × 7)	1	32 × 32 × 32
4	Residual block 2	64 filters size of (3 × 3 or 5 × 5 or 7 × 7)	1	32 × 32 × 64
5	Residual block 3	128 filters size of (3 × 3 or 5 × 5 or 7 × 7)	1	32 × 32 × 128
6	Dense layer 1	1,024 units with dropout rate: 0.2	–	1024
7	Dense layer 2	512 units with dropout rate: 0.2	–	512
8	SoftMax	–	–	4 or 2

Let the number of EEG feature *S*_*i*_(*i* = 1,…,*M*) used for model training is *M*, and the total number of label categories is *C*, the loss function of the classifier is denoted by *L*_*y*_, whose formula is:


(6) $$L_{y}\,\left(S_{i};\theta_{f},\theta_{y}\right)=\sum_{i=1}^{M}\sum_{y=1}^{C}-\phi \,\left(l_{i},y\right)\times \log P\,\left(y|S_{i}\right)$$

where θ_*f*_ and θ_*y*_ represent the parameters of the feature extractor and the label predictor, respectively. *l*_*i*_ represents the true label of the sample *S*_*i*_, and ϕ(*l*_*i*_,*y*) is expressed as,


(7) $$\varphi \,\left(l_{i},y\right)=\left\{ \begin{array}{c} 1,i\,f\,l_{i}=y,\\ 0,o\,t\,h\,e\,r\,w\,i\,s\,e \end{array}\right.$$

By minimizing the loss function *L*_*y*_(*S*_*i*_;θ_*f*_,θ_*y*_), the emotion category of each training EEG sample can be predicted accurately to the maximum extent. Let *S*_*test*_ denote the test set, and *l*_*test*_ denote the probability of predicted labels on the test set which can be calculated as the following formula:


(8) $$l_{t\,e\,s\,t}=\arg\max_{c}\left\{P\left(c|S_{t\,e\,s\,t}\right)|c=1,\ldots ,C\right\}$$

#### Optimization of Residual Convolutional Neural Network Model

**FIGURE 5:**
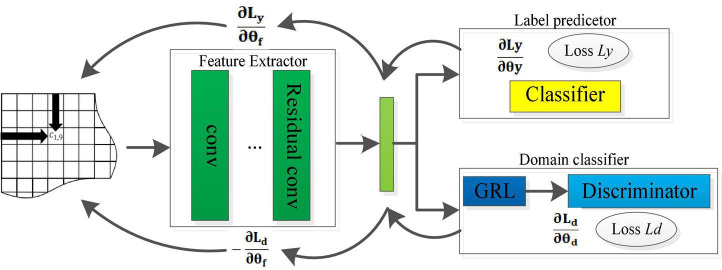
Structure of the optimized DA-RCNN model.

When making EEG-based emotion recognition, it should be noted that the EEG samples for training and testing may come from different subjects or from different trails of the same subject, which leads to poor adaptability and robustness of the model. To solve this problem, we introduce a domain discriminator in the RCNN model, which works jointly with the feature extractor and the classifier to learn more emotionally discriminative domain invariant features. This optimized domain adaption RCNN model (DA-RCNN) is shown in Figure 5, which demonstrates the domain adaptive optimization process of the whole model.

Specifically, assuming two EEG datasets $D^{S}=\left\{S_{1}^{S},\ldots ,S_{M_{1}}^{S}\right\}$ and $D^{T}=\left\{S_{1}^{T},\ldots ,S_{M_{2}}^{T}\right\}$ are obtained from the source domain (training set) and the target domain (test set), where *M*_1_ and *M*_2_ represent the number of samples in the source dataset and target dataset, respectively. To alleviate the domain difference, the discriminator loss function is defined as,


(9) $$L_{d}\,\left(S_{i}^{S},S_{j}^{T};\theta_{f},\theta_{d}\right)=-\sum_{i=1}^{M_{1}}\log P\,\left(0|S_{i}^{S}\right)-\sum_{j=1}^{M_{2}}\log P\,\left(1|S_{j}^{T}\right)$$

Where $P\,\left(0|S_{i}^{S}\right)$ represents the probability that the sample $S_{i}^{S}$ belongs to the source domain, $P\,\left(1|S_{j}^{T}\right)$ represents the probability that the sample $S_{j}^{T}$ belongs to the target domain, and θ_*d*_ represents the parameters of the domain discriminator. By maximizing the loss function, the feature learning process will gradually generate the domain invariant features between the training set and the test set in emotion recognition.

As known, by minimizing the loss function (6) of the classifier, the emotion category can be predicted on the test set with the model trained on the training set. Through maximizing the loss function (9) of the discriminator, better domain invariance features can be learned so that the model has better adaptability and robustness. Therefore, we define the loss function of the whole DA-RCNN model as,


(10) $$L\,\left(S^{S},S^{T}|\theta_{f},\theta_{c},\theta_{d}\right)=L_{y}\,\left(S^{S};\theta_{f},\theta_{y}\right)-L_{d}\,\left(S^{S},S^{T};\theta_{f},\theta_{d}\right)$$

Our goal is to find the best parameters that minimize formula (10) by minimizing *L*_*y*_(^*SS*^;θ_*f*_;θ_*y*_) and maximizing *L*_*d*_(^*SS*^,^*ST*^;θ_*f*_,θ_*d*_) through synchronous iterations. To use a stochastic gradient descent algorithm to find the optimal model parameters, we introduce a gradient reverse layer (GRL) to make the discriminator convert the maximization problem into a minimization problem. The GRL performs consistent conversion during the forward propagation process but performs gradient sign inversion during the back propagation process. At this point, the parameter updating process can be expressed as (Where α represents the learning rate),


(11) $$\theta_{d}\leftarrow \theta_{d}-\alpha \,\frac{\partial L_{d}}{\partial \theta_{d}},\theta_{f}\leftarrow \theta_{f}+\alpha \,\frac{\partial L_{d}}{\partial \theta_{f}}$$

## Experiments and Results

In this part, we will make subject-dependent and subject-independent emotion recognition experiments with the proposed model on the DEAP dataset and show the experimental results. The classification accuracy is used to evaluate the performance of the models. Through making a lot of comparative experiments, we systematically analyze and discuss the impact of different components of the proposed model on the overall recognition performance.

### Dataset

The DEAP dataset (Koelstra et al., 2012) is currently the most widely used large-scale public sentiment analysis database of physiological signals. It records the physiological signals of EEG, EOG, EMG, and other physiological signals induced by 32 subjects watching 40 music videos with different emotional tendencies for about 1 min. Each subject evaluated the arousal, valence, preference, dominance, and familiarity of each video using a continuous numerical value of 1–9. We take 32-channel EEG recordings of each subject for 63 s collected by the Bio-Semi system from the DEAP data set as the research data. The electrodes of 32-channel EEG data are positioned according to the 10–20 system (Oostenveld and Praamstra, 2001), and the EEG recordings are down sampled to 128 Hz. To eliminate DC noise and other artifacts, a 4∼45 Hz band-pass filter is used for data filtering, and the blind source separation technology is then used to remove the electrooculogram interference.

### Data Preprocessing and Feature Extraction

In the DEAP data set, the original EEG signal is represented as 32(subs) × 40(trials) × 32(channel) × 8,064(samples), where 8064 = 128(time points/s) × 63(s), and the label is represented as 40(trials) × 4. Due to the human visual response delay, we take the EEG signals of the first 3 s as the benchmark, and the subsequent 60 s of the EEG signals are used as experimental data. Thus, the preprocessed data is formatted as 32(subs) × 40(trials) × 32(channels) × 7680(points). The first two dimensions of valence and arousal evaluation values are used to generate the emotional labels, whose size is 40(trials) × 2.

In the process of data preprocessing, we use the window length of 1, 2, and 3 s to segment the EEG sequence and make the experiment, and the experimental results have found that the performance is better when the window length is 3 s. In order to obtain more samples and complete BC information, we set the window moving step as 0.5 s, that is, using the overlap of 2.5 s to segment the EEG sequence across all 32 channels. As a result, we get 115 segments and each segment contains 384 time points. Therefore, the EEG data of each subject is formatted as 40 × 32 × 384 × 115. Then, the data format is converted to 32 × 384 × 4,600, which means that each subject contains 4,600 samples in the subject-dependent experiment. In a subject-independent experiment, the total number of samples is 1,47,200 (4600 × 32 subjects), and the size of each sample is 32(channels) × 384(time points) × 1,47,200. Next, the labels of EEG data are obtained based on the subjects’ emotional evaluation values in the range of 1–9 in valence and arousal dimensions. The median value of 5 is used as the threshold to divide the evaluation values into two categories. The values greater than 5 represent the high valence or arousal category and are labeled by 1. The values less than or equal to 5 represent the low valence or arousal category and are labeled by 0. Because the number of trials corresponding to different labels is basically the same, there is no need to perform sample balancing.

Then, the PSD feature of each EEG sample is extracted, based on which the global brain network connectivity feature matrix is constructed with four algorithms of PCC, PLV, TE, and WCC, respectively. This process converts two-dimensional PSD features into the connectivity feature matrix carrying spatiotemporal features, and the feature size of each sample is converted from 32 × 384 to 32 × 32. In the process of feature extraction and conversion, the label size is changed synchronously with the number of samples. Table 1 demonstrates the EEG data and labels the shape of different features in two scenarios.

### Benchmark Models

To verify the effectiveness and advantages of the proposed model, we apply 2 classical machine learning algorithms [BT (Chuang et al., 2012) and SVM (Suykens and Vandewalle, 1999)] and 4 deep neural networks [LSTM (Alhagry et al., 2017), DBN (Zheng and Lu, 2015), CNN (Chen et al., 2019b), BiLSTM] as benchmark models. The subject-dependent and subject-independent emotion recognition experiments are carried out on the same DEAP data set. The best connectivity matrix feature and the most widely used PSD feature are selected as the input of the benchmark models to explore the advantages of the proposed feature and model. We separately fine-tune the parameters of different benchmark models to ensure they all work out their best performance to make a fair comparison. Table 2 shows the fine-tuned hyperparameters of each given benchmark model.

### Experimental Results and Analysis

In the experiment, we verify the accuracy of the proposed method in two-class emotion recognition in terms of valence and arousal in both subject-dependent and subject-independent scenarios. In the subject-dependent scenarios, both the training data and the test data come from the different trials of the same subject. That is, 4 (about 10%) trials that were randomly selected from the EEG data of the same subject each time are used as the test set, and the samples of the remaining 36 trials are used as the training set. In this way, a 10-fold cross-validation set is constructed for each subject. The average of the accuracy on the 10 test sets is taken as the recognition accuracy of each subject. In the subject-independent scenario, we adopt the Leave-one-subject-out (LOSO) cross-validation strategy to extract the EEG data of one subject as the test set in each fold, and the rest of the subjects’ EEG data is used as the training set. The classification accuracy of 32 subjects in the two scenarios was averaged as the final recognition result.

#### Experimental Results of the Proposed Method

This article uses the TensorFlow framework to train the model from scratch in a fully supervised manner based on NVIDIA Titan X Pascal GPUs. The choice of hyperparameters is very important for deep learning models, as a good set of hyperparameters can improve the prediction performance of deep models. In order to obtain the optimal hyperparameters of the proposed model, four different residual blocks (*r* = 1, *r* = 3, *r* = 5, *r* = 7) and 2 residual convolution kernels of different sizes (3 × 3, 5 × 5) are, respectively, used to build the RCNN model to evaluate the effect of the number of residual blocks and the size of the residual kernel on the performance. For the convenience of comparison, the DA-RCNN model was used to conduct subject-dependent binary valence and arousal classification experiments on the PLV connectivity features. Table 3 lists the classification results of the DA-RCNN model configured with different hyperparameters, where *Sp* means specificity, *Sn* means sensitivity, and *Acc* means accuracy. When the residual block is 3 and the kernel size is 5 × 5, it has the highest sensitivity and accuracy compared with other settings, that is 98.42 and 95.15% in valence, and 98.90 and 94.84% in the arousal, respectively. In this case, the classification performance of the model is optimal. It is further found that with the increase in the number of residual blocks, the overall performance of the model is increasing. When the number of residual blocks is 3, the overall performance of the model reaches the optimum. However, with the increase of the number of residual blocks, the classification performance decreases, which may be because the binary emotion classification task is relatively simple. If complex classification problems are faced or the classification types reach hundreds or even thousands, the classification performance may be improved with the increase in residual blocks.

Therefore, the DA-RCNN model with 3 residual blocks and different sizes of residual kernels (3 × 3, 5 × 5, 7 × 7) is, respectively, used to make emotion classification experiments on the five features of PSD, PCC, PLV, TE, and WCC to verify the effectiveness and superiority of the brain network connectivity features. The input, output, data format, and optimal hyper parameters of each layer of the model are shown in Table 4.

The experimental results are shown in Table 5. As seen in Table 5, in the subject-dependent scenario, the DA-RCNN model with the residual kernel size of 5 × 5 gets the best valence and arousal binary emotion classification accuracy of 95.15 and 94.84% on the PLV feature, which is 2.78 and 2.79% higher than the accuracy on the suboptimal PCC feature, is 5.75 and 4.94% higher than that on the WCC feature and is 7.23 and 7.19% higher than the accuracy on the common PSD feature, respectively. In the subject-independent scenario, the DA-RCNN model with a residual kernel size of 5 × 5 also gets the best valence and arousal emotion classification accuracy of 88.28 and 87.66% on the PLV feature, which is 2.86 and 3.55% higher than that on the suboptimal PCC feature, is 4.54 and 5.1% higher than that on the WCC feature, and is 7.82 and 8.1% higher than that on the common PSD feature, respectively. In conclusion, the classification performance of the DA-RCNN model on the PLV connectivity feature is higher than that on the PCC, TE, and WCC features, respectively. It proves that the PLV connectivity feature can better estimate the instantaneous phase-locked relationship between different channels of EEG, and the spatial arrangement and phase information of EEG electrodes help better predict the emotional tone and achieve better emotion recognition accuracy. Moreover, the classification performance on the three connectivity features is all better than that of the common PSD feature, and this advantage is more obvious in the subject-independent scenario, which shows that when recognizing EEG signals with domain heterogeneity, the PSD feature processed by the brain network connectivity algorithms is more discriminative than the common PSD feature. It also verifies the effectiveness of the proposed domain adaptive residual convolutional network on BC features for emotion classification.

**TABLE 5:** Overall performance of the proposed DA-RCNN model on different features.

Models		Features	Subject-dependent		Subject-independent	
			Valence acc (%)	Arousal acc (%)	Valence acc (%)	Arousal acc (%)
DA-RCNN	w = 3 × 3	PSD	87.12	86.90	79.20	78.40
	w = 5 × 5		87.92	87.65	80.46	79.50
	w = 7 × 7		85.40	84.10	78.50	78.95
	w = 3 × 3	PCC	89.06	89.14	84.09	83.51
	w = 5 × 5		92.37	92.05	85.42	84.05
	w = 7 × 7		90.50	89.42	82.17	81.36
	w = 3 × 3	PLV	93.25	92.95	86.05	85.15
	w = 5 × 5		**95.15**	**94.84**	**88.28**	**87.60**
	w = 7 × 7		92.37	92.05	85.73	84.98
	w = 3 × 3	TE	88.14	88.73	80.08	80.14
	w = 5 × 5		89.06	89.73	81.50	81.39
	w = 7 × 7		87.17	87.82	79.44	79.47
	w = 3 × 3	WCC	88.09	89.32	81.18	81.09
	w = 5 × 5		89.40	89.90	83.74	82.20
	w = 7 × 7		89.72	87.61	82.33	80.82

*Bold values represent the best comparative results.*

It is also found that the model with the residual kernel of 5 × 5 performs better than the model with the kernel of 3 × 3. For example, in a subject-independent scenario, the classification performance is increased by 2.23 and 2.45% in valence and arousal, respectively. It shows that convolution on a relatively large area can capture more emotion-related information, but the residual kernel of 7 × 7 does not further improve the performance of the model, which is probably due to the increase of model parameters and excessive smoothing caused by the larger convolution kernel.

#### Comparison With Benchmark Methods

To show the superiority of the proposed method, the six benchmark methods described in section “Benchmark Models” are used for comparative experiments. The above experimental results have shown that the DA-RCNN model with a residual kernel of 5 × 5 performs better on the PLV feature. Therefore, the standard PSD feature and the PLV connectivity feature are selected as the input of various models for comparison. Furthermore, we make paired *t*-tests against all the comparative models to evaluate their statistical differences in emotion classification performance on the same type of EEG features. For all paired *t*-tests, *p-*value correction was performed for multiple hypothesis tests using the Bonferroni criterion and implementation method to limit the error rate (FDR). *p-*values represent corrected results of paired *t*-tests. *p* < 0.05 indicates the difference is significant. The experimental and statistical test results are shown in Table 6.

**TABLE 6:** The emotion classification accuracy (%) comparison of various methods.

Models	Features	Subject-dependent		Subject-independent	
		Valence acc (*p*-value)	Arousal acc (*p*-value)	Valence acc (*p*-value)	Arousal acc (*p*-value)
BT	PSD	78.65 (0.0061)	78.18 (0.0059)	70.67 (0.0052)	71.33 (0.0048)
	PLV	80.13 (0.0027)	80.50 (0.0031)	73.98 (0.0040)	73.06 (0.0043)
SVM	PSD	79.75 (0.0043)	78.90 (0.0040)	70.92 (0.0015)	71.20 (0.0016)
	PLV	80.62 (0.0014)	80.15 (0.0021)	75.14 (0.0020)	74.90 (0.0029)
DBN	PSD	81.50 (0.0006)	81.30 (0.0011)	75.37 (0.0049)	75.45 (0.0052)
	PLV	83.12 (0.0007)	82.76 (0.0008)	77.10 (0.0050)	77.67 (0.0032)
LSTM	PSD	82.61 (0.0033)	81.95 (0.0041)	76.80 (0.0014)	77.14 (0.0010)
	PLV	85.66 (0.0009)	85.90 (0.0013)	80.85 (0.0010)	80.25 (0.0003)
CNN	PSD	83.03 (0.0043)	83.25 (0.0039)	78.83 (0.0032)	78.95 (0.0039)
	PLV	87.60 (0.0058)	86.50 (0.0062)	81.10 (0.0060)	80.60 (0.0049)
BILSTM	PSD	85.12 (0.0010)	84.19 (0.0012)	79.75 (0.0073)	80.12 (0.0065)
	PLV	90.71 (0.0006)	90.27 (0.0007)	83.89 (0.0045)	82.75 (0.0050)
DA-RCNN (w = 5 × 5)	PSD	88.66	87.65	82.95	79.50
	**PLV**	**95.15**	**94.84**	**88.28**	**87.60**

*Bold values represent the best comparative results.*

From the results of the subject-dependent experiment, in the valence dimension, the classification performance of BT, SVM, DBN, LSTM, CNN, BiLSTM, and DA-RCNN models on the PLV feature is 1.48, 0.87, 1.62, 3.05, 4.57, 5.59, and 6.49% higher than that on standard PSD feature, respectively. In the arousal dimension, the classification performance of BT, SVM, DBN, LSTM, CNN, BiLSTM, and DA-RCNN models on PLV feature is 2.32, 1.25, 1.46, 3.95, 3.25, 6.08, and 7.19% higher than that on standard PSD features, respectively. It shows that the proposed PLV connectivity feature accurately carries more emotion-related information than the standard PSD feature, and the spatial connectivity information of the brain network is more effective for emotion recognition. Compared with the benchmark models, in valence dimension, the proposed DA-RCNN model outperforms the suboptimal deep learning BiLSTM model by 2.8 and 4.44% on PSD and PLV features, respectively, outperforms the better shallow SVM model by 8.42 and 14.53% on PSD and PLV features, respectively, and the differences are all significant, indicating that the deep learning model has stronger feature learning ability than the shallow model, and the proposed model can more accurately extract the emotion-related high-level information from EEG, especially from the brain network connectivity features than the other comparative models. Moreover, the classification accuracy of the proposed DA-RCNN model on the PLV feature is 12.03, 9.49, 7.55, and 4.44%, respectively, higher than that of the DBN, LSTM, CNN, and BiLSTM models in valence and is, respectively, 12.08, 8.94, 8.34, and 4.57% higher than that of the DBN, LSTM, CNN, and BiLSTM models in arousal, and the differences are all significant, which verifies the effectiveness and superiority of the proposed model.

From the results of the subject-independent experiment, we can show that the classification accuracy of the proposed DA-RCNN model on the PLV feature is, respectively, 11.18, 7.43, 7.18, and 4.39% higher than that of DBN, LSTM, CNN, and BiLSTM models in valence and is, respectively, 9.93, 7.35, 7, and 4.85% higher in arousal, and the differences are all significant, which further verifies the effectiveness and superiority of the proposed DA-RCNN model in emotion recognition.

We calculate the parameters of several comparative models to discuss the size and the computational efficiency of the proposed model. The total number of parameters of the proposed model is 67,088, while under the same experimental settings, the parameters of the benchmark models CNN, LSTM, DBN, and BiLSTM are 31,042, 30,866, 40,282, and 50,156, respectively. Although the number of parameters and the training time of the proposed model is larger and longer than that of the comparative model, the classification accuracy is significantly improved.

#### Model Training Process

**FIGURE 6:**
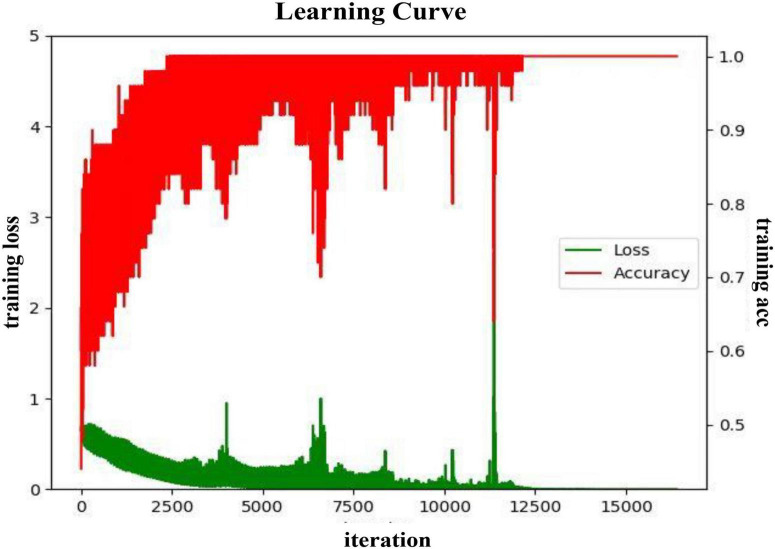
The training process curve in the subject-dependent experiment in the valence.

**FIGURE 7:**
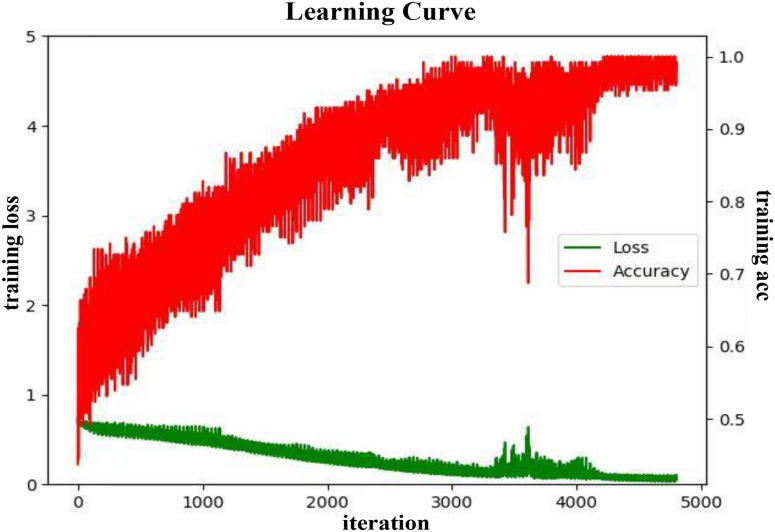
The training process curve in the subject-independent experiment in the valence.

In the valence dimension, the training process of the proposed DA-RCNN model on the PLV feature in the subject-dependent and subject-independent scenarios are shown in Figures 6, 7, respectively. In both figures, the green line represents the average training loss, the red line represents the average training accuracy (acc), the horizontal axis represents the number of iterations, the training loss value refers to the left vertical axis, and the training accuracy value refers to the right vertical axis.

It can be seen from Figure 6 that in the subject-dependent experiment, the average loss of the model during the training process is gradually decreasing and converging, and the training accuracy rate is also gradually converging to 1. During the iterations from 0 to 3,000 rounds, the loss value declines in a spiral gradient downward trend, and the accuracy increases from about 0.5–0.93 in a spiral gradient upward trend. Subsequently, both the loss value and the accuracy rate oscillate to a certain extent, but after 9,000 rounds of iterations, the amplitude became significantly smaller, and finally, the training loss converged to 0 and the accuracy rate converged to 1. In this training process, the learning rate of the model is finally selected as 0.001, the batch size is 40, and the epochs are 200.

It can be seen from Figure 7 that in the subject-independent experiment, during the training process, the training loss of the model gradually converges to 0, while the training acc spirals upward and converges to 1. When the iterations are around 3,500, there is a large fluctuation but then a stable trend in the loss. In this area, the accuracy also has a large change in the same trend. It may be because the gradient falls into the local optimal solution and keeps swaying during two ends of the correct gradient. As the training data is updated, the Adam optimizer continues to correct the parameters by bias. In this training process, the learning rate is finally selected as 0.005, the batch size is 128, and the epochs is 150.

During the fine-tuning process, it is found that when the initial learning rate is too large, the gradient continuously sways at the left and right ends of the correct gradient, which will cause local oscillations and make the model fail to converge. When the learning rate is set too small, the model will be more complex, and more parameters need to be updated in each iteration. If the input data is insufficient, the loss function will oscillate and not converge. Therefore, only when the learning rate is within a reasonable range, the learning of the model and the updating of the parameters can be carried out effectively.

**FIGURE 8:**
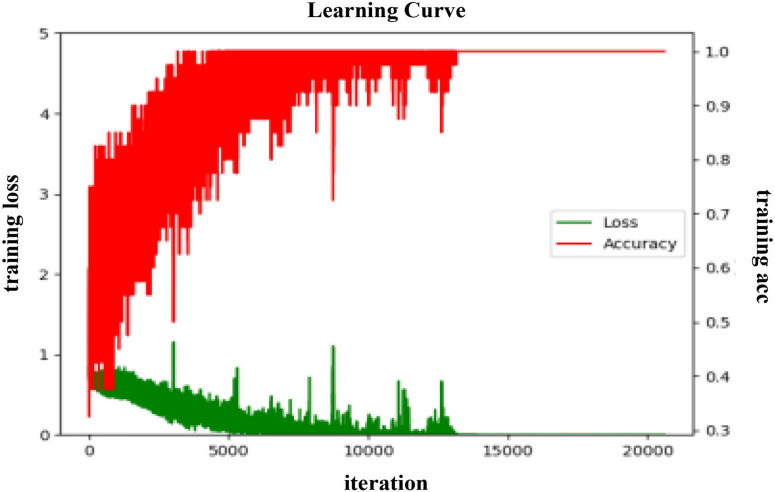
The training process curve in the subject-dependent experiment in arousal.

**FIGURE 9:**
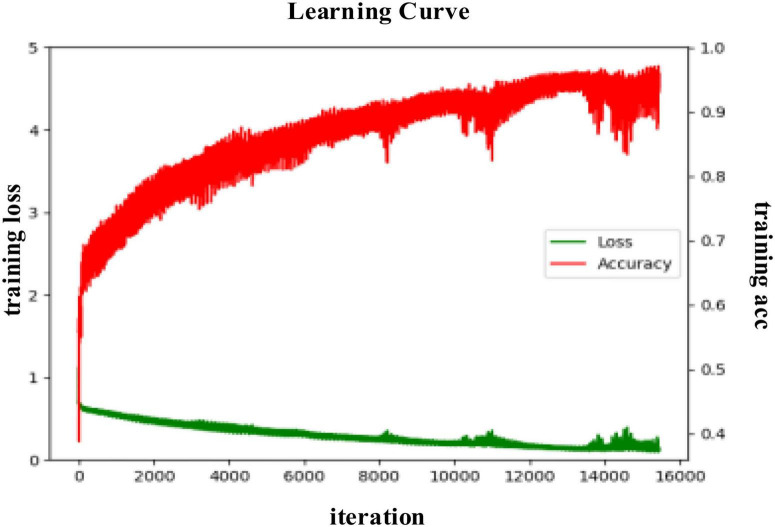
The training process curve in the subject-independent experiment in arousal.

In the arousal dimension, the training process of the BC-DA-RCNN model on the PLV BC features in subject-dependent and subject-independent scenarios is shown in Figures 8, 9, respectively.

As seen from Figure 8, during the training process of the proposed model in a subject-dependent experiment in arousal, the average error of the model is gradually decreasing and converging, and the training accuracy rate is gradually converging to 1. During the period from 0 to 5,000 iterations, the error value shows a downward spiral gradient, and the decline rate is fast and stable. The accuracy during this period increases from about 0.48% to around 0.97% in a spiral gradient upward trend. In the subsequent training process, the error value and the accuracy rate both fluctuate to a certain extent, but after 10,000 rounds, the fluctuation becomes significantly smaller, and then there are two sharp upward trends, and then quickly decrease, but it is believed to be a normal phenomenon due to the fact that the gradient during the learning process is not the optimal direction of the objective function. As the iteration continues, eventually the training error converges to 0 and the accuracy converges to 1.

It can be seen from Figure 9 that during the training process of the proposed model in a subject-independent experiment in arousal, the training loss decreases gradually and finally converges to 0, the parameters are updated toward better performance and the training accuracy spirals upward and converges to 1. Compared with the process in valence, the training process in arousal is gentler, which may be related to the data distribution differences. When the number of iterations reaches 14,000, there is a large oscillation, probably because the local optimum is reached, but as the learning continues and the parameters are updated and optimized, the gradient drops in the correct direction.

## Discussion

### Performance Analysis of Different Variants of the Proposed Model

It has been proven that the proposed DA-RCNN model achieves the best performance in both subject-dependent and subject-independent experiments. It largely attributes to our method that not only applies the spatial connectivity information of the brain network but also uses the domain-adaptive residual convolution neural network to extract the highly discriminative high-level features from that. To verify this point, we obtain the following 3 simplified variants by removing the BC feature and the domain adaptation layer:

(1) RCNN: Both the brain network connectivity feature and the domain adaptation layer are removed, leaving only the RCNN model to learn from the input of the standard PSD feature.
(2) DA-RCNN: Only the brain network connectivity feature is removed, and the standard PSD feature is used as the input to the model;
(3) BC-RCNN: Only the domain adaptation layer is removed, that is the domain transfer of features between subjects is neglected, and the optimal PLV connectivity feature is used as the input to the model.

The performance comparison of these three variants in subject-dependent and subject-independent binary valence classification experiments is shown in Table 7, and the overall performance comparison relationship is as follows:

**TABLE 7:** The experimental results of three variants of the proposed model.

Methods	Subject-dependent		Subject-independent	
	Valence acc (%)	Arousal acc (%)	Valence acc (%)	Arousal acc (%)
RCNN	85.50	85.11	80.98	80.70
DA-RCNN	88.66	87.03	82.95	82.55
BC-RCNN	90.59	90.05	85.60	85.13
BC-DA-RCNN	**95.15**	94.84	**88.28**	**87.38**

*Bold values represent the best comparative results.*


R⁢C⁢N⁢N<D⁢A-R⁢C⁢N⁢N<B⁢C-R⁢C⁢N⁢N

(12) <B⁢C-D⁢A-R⁢C⁢N⁢N

It can be seen from Table 7, that in two scenarios, the classification accuracy of the RCNN model is 0.35 and 0.82% higher in valence and 0.92 and 0.58% higher in arousal than the best benchmark BiLSTM model in Table 6, which indicates that skip connections and cascaded residual convolutions operations in RCNN playing a very important role in feature learning and outperforming other models. Compared with DA-RCNN, the classification accuracy of the proposed model is 6.49 and 5.33% higher in valence, and 7.81 and 4.83% higher in arousal, indicating that the brain network connectivity information contains richer emotion-related features, and the connectivity measurement algorithms, such as PLV, PCC, WCC, and TE, can effectively represent the brain network connectivity gain information, thereby helping to improve the performance of emotion recognition. Compared with BC-RCNN, the classification accuracy of the proposed model is 4.56 and 2.68% higher in valence, and 4.79 and 2.25% higher in arousal, respectively, indicating that the DA layer does help to extract more emotion-discriminative and subject-independent EEG features and improve the adaptability of the model. Compared with the DA-RCNN model, the classification accuracy of the BC-RCNN model in the two experiments is 1.93 and 2.65% higher in valence, and 3.02 and 2.58% higher in arousal, respectively, indicating that the BC feature is more critical to the overall performance of the proposed method. All in a word, the proposed BC-DA-RCNN method outperforms the existing other methods and achieves better EEG-based emotion recognition performance with less signal processing and a simpler model structure.

### Discussion of Electrode Arrangement Mode

The order of EEG electrodes plays an important part in constructing the connectivity matrix and affecting the performance of the feature. Furthermore, the connectivity feature matrix must be robust to the task-independent fluctuation of EEG signals. We use the physical distance between EEG electrodes to determine the alignment order of the electrodes, whose mode is denoted by “dist-mode” and is not necessarily optimal. To test the influence of the EEG electrode arrangement mode on the classification performance, we cite the algorithm mentioned in the literature (Chen and Wang, 2019), which uses a one-dimensional scaling (UDS) algorithm to determine the electrode driving mode with global and local features. The UDS algorithm can project the given multidimensional data into a one-dimensional mapping space while preserving the relative distance between channels as much as possible. This distance is defined by the disparity function. The resulted global and local electrode arrangement mode is called “global-mode” and “local-mode,” respectively. The global mode can obtain the electrode driving order that enhances the global feature, and its disparity function is defined as formula (13):


(13) $$\delta \,\left(i,j\right)=2\,\left(1-c_{i,j}\right)$$

where *c*_*i*,*j*_ represents the connectivity measure between the *i*-th and *j*-th electrodes. This disparity function is inserted into the objective function of UDS, which was called the normalized stress function (De Leeuw, 1977) and written as:


(14) $$s\,t\,r\,e\,s\,s\,\left(l_{1},\ldots ,l_{N\,e}\right)=\frac{\sum_{i<j}{\left(\left|l_{i}-l_{j}\right|-\delta \,\left(i,j\right)\right)}^{2}}{\sum_{i<j}\delta \,{\left(i,j\right)}^{2}}$$

where |*l*_*i*_−*l*_*j*_| represents the Euclidean distance between the *i*-th and *j*-th electrodes in the projected space. The continuous solution value *l*_1_,…,*l*_*Ne*_ is obtained by minimizing the objective function so that the value of the disparity function is as similar as the distance in the one-dimensional projected space on average. Then we discard the distance information and only keep the order of the solutions, in which the EEG electrodes are arranged in horizontal and vertical directions of the connection matrix.

The local mode can obtain the electrode driving order that enhances the local feature, and its disparity function is defined as formula (15):


(15) $$\delta \,\left(i,j\right)=c_{i,j}^{2}$$

Again, the normalized stress function (14) using this disparity function is minimized and the order information of the solution is obtained. In the connectivity matrix arranged in this order, the brain regions with strong positive or negative connectivity are separated as much as possible, and the regions with zero correlation are arranged as close as possible, thereby enhancing the local characteristics of the brain network.

Table 8 shows the valence classification results of the proposed model on PCC and PLV connectivity features expressed with different connectivity modes (the TE feature is not included here). It can be seen that in two experiments, when the residual kernel of the proposed model is 5 × 5 and on the PLV feature, the classification accuracy, respectively, achieves 94.31 and 87.22% with global connectivity mode, which is very close to the performance with the proposed global dist-mode but is, respectively, 6.75 and 4.54% higher than the performance with the local connectivity mode. It shows that the global driving mode is better than the local mode to obtain the brain network features, and the connectivity features generated with the global arrangement contain more emotion-related context information that is exactly extracted by a deep residual convolutional neural network. Meanwhile, it also proves the effectiveness of our global connectivity feature based on the proposed physical distance arrangement. The reason why the global mode is better than the local mode may be that the local mode fails to fully consider the spatial connectivity gain information of all EEG electrodes. It further confirms that human emotion is the result of the interaction of different areas of the whole brain, and the global brain network feature is more comprehensive and effective. In addition, the result in Table 8 also shows that the proposed model has better classification performance on the PCC and PLV features when the residual kernel is 5 × 5, which is consistent with the experimental results in section “Experimental Results of the Proposed Method.”

**TABLE 8:** The classification results using different connectivity mode.

Experiment type	Connectivity mode	PCC (Accuracy %)		PLV (Accuracy %)	
		w = 3 × 3	w = 5 × 5	w = 3 × 3	w = 5 × 5
Subject-dependent	Dist-mode	92.05	92.37	93.25	**95.15**
	Global-mode	90.56	91.28	93.15	**94.31**
	Local-mode	84.41	84.79	86.95	**87.56**
Subject-independent	Dist-mode	86.42	86.05	87.60	**88.28**
	Global-mode	85.64	86.70	86.64	**87.22**
	Local-mode	81.07	82.19	81.27	**82.68**

*Bold values represent the best comparative results.*

### Discussion on Electroencephalograph Connectivity Features

**FIGURE 10:**
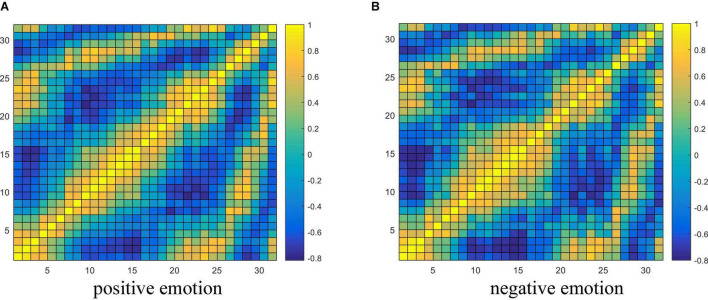
Visualization of PCC connectivity features. **(A,B)** Respectively shows the average PCC connectivity pattern under the positive and negative emotion.

**FIGURE 11:**
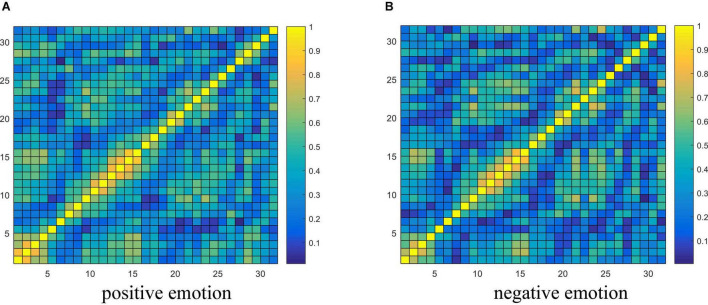
Visualization of PLV connectivity features. **(A,B)** Respectively shows the average PLV connectivity pattern under the positive and negative emotion.

From the experimental results in section “Performance Analysis of Different Variants of the Proposed Model,” it is concluded that the brain network connectivity information is the key to improving the final classification performance of the model. In this section, the connectivity values of all subjects related to each type of emotion are averaged and visualized to interpret what connections are more important for emotion recognition and how the connectivity patterns are associated with the emotional processing function of the brain. Due to the poor performance of the TE feature, only the visualization of PCC and PLV connectivity features are presented here. The horizontal and vertical axes both represent the electrodes, and the electrode sequence here is obtained by the proposed data driving method as shown in Figure 2. The average PCC connectivity matrix values are between −1 and 1, and its average connectivity patterns under positive and negative emotions are shown in Figures 10A,B, respectively. The average PLV connectivity matrix values are between 0 and 1, and its connectivity patterns under positive and negative emotions are shown in Figures 11A,B, respectively.

It can be seen from Figure 10 that the bright region represents an area with a large Pearson coefficient, and the dark region represents an area with a small Pearson coefficient. Comparing the electrode positions in Figure 2, we can see that each electrode has strong connectivity with the three electrodes before and after its own position, which proves the effectiveness of the proposed electrode driving method. It was also observed that the Pearson correlation coefficient between the 20th and 25th electrodes (belonging to the right frontal lobe) and the 1st and 4th electrodes (belonging to the left frontal lobe) was larger for both positive and negative emotions, indicating there are a stronger correlation and more significant neuronal activity between the left and right frontal areas of the brain, and the connectivity of the frontal lobe area is helpful for emotion recognition. In addition, there is a strong correlation between the 26th and 29th electrodes (belonging to the parietal lobe region) and the 10th and 15th electrodes (belonging to the occipital lobe region), indicating that the parietal and occipital regions are more sensitive to the emotional activity of the brain, and it is easy to extract emotional-related connectivity information from this area. The brightness between electrodes 31th and 32th (belonging to the parietal lobe region) and 1st and 3rd (belonging to the left frontal region) and electrodes between 21th and 25th (belonging to the right frontal region) was also higher, which further verified the above analysis.

Figure 11 shows that the bright region represents an area with a larger phase-lock value, and the dark region represents an area with a smaller phase-lock value. Compared with the electrode positions in Figure 2, we can that each electrode has strong connectivity with its adjacent two electrodes before and after its own position, which verifies the effectiveness of the proposed electrode driving method. It was further found that the phase synchronization index between electrodes 1st and 5th (belonging to the left frontal region) and between electrodes 10th and 15th (belonging to the occipital lobe region) was larger, indicating that when evoking positive and negative emotions, the synergy between the left frontal and occipital regions of the brain is enhanced, resulting in synchronous oscillations. Through the contrast of the light and dark in the regions where other electrodes are located, it is found that the phase consistency of evoked emotional tendency information in the frontal, parietal and occipital regions is relatively strong, whereas the phase consistency in other regions is poor.

### Limitation Discussion

The paper for the first time proposes to combine the brain network connectivity features with the RCNN to construct a high-performance and low-complexity emotion recognition model and additionally introduces a DA module to enhance the model’s adaptive ability. The proposed method has achieved good performance in the binary emotion classification task, but there are still the following limitations that need to be addressed in our future work:

#### Insufficient Exploration of the Features of Brain Network Connectivity

Existing research have proposed a variety of methods to explore brain network connectivity. Although we apply three connectivity measurement algorithms (PCC, PLV, and TE) and prove they are effective and better, it does not fully prove which method is best. Taking the TE feature as an example, we directly use the first-order TE feature without considering the time delay. It is expected to further improve the performance of the TE feature if more freedom of parameter selection can be provided. Moreover, the judgment of connectivity not only depends on the choice of different connectivity measurements but also depends on the dimension of thinking. For example, a recent study (Li R. et al., 2021) utilizes the topology of EEG channels to measure the BC and applies an adaptive graph convolution network (MD-AGCN) to learn the deeply fused intra-channel and inter-channel time-frequency information. In the future, we will deeply study the cognitive mechanism of the brain network and explore the best representation of BC features to further improve the performance of EEG-based emotion recognition.

#### Deficiencies of Fully Supervised Training Mode

Another problem is that the proposed model adopts a fully supervised training mode, which solves the global optimization problem facing neural networks through backpropagation but is difficult to meet the complex real-time application requirements. On the contrary, the self-supervised method does not rely on many hand-annotated labels and can use information from the dataset itself to fake labels and has more convincing learning potential than a completely unsupervised method. Starting from the nature of human learning, the self-supervised method can make self-learning with a small amount of annotated data to realize spontaneous learning from unmarked data sets. In the future, we will study to build a self-supervised model to make EEG-based emotion recognition and meet the needs of real-time applications of affective brain-computer interfaces.

## Conclusion

The study proposes an end-to-end emotion recognition method based on EEG connectivity features and a domain adaptive residual convolution neural network. Four classical connectivity metric algorithms are used to measure the brain network connectivity between different EEG electrodes and construct the connectivity feature matrix. The proposed adaptive residual convolution model is optimized by introducing the residual blocks and the domain adaptation module to a CNN model. Various comparative experiments are carried out to prove the effectiveness and advantages of the proposed method. The experimental results show that the PLV and PCC connectivity features contain rich emotion-related information that is not obvious in original EEG signals. It further confirms that the brain network connectivity matrix can reflect the human’s emotional dynamics to a certain extent, which provides a new and effective way for EEG feature representation. It also proves that the DA-RCNN can learn more emotion-discriminative and subject-independent high-level information from PLV and PCC connectivity features, which help further improve the accuracy and stability of EEG emotion recognition. Meanwhile, the complexity of the model is greatly reduced through the residual block structure, which is beneficial for real-time applications in affective brain-computer interfaces.

## Data Availability Statement

The original contributions presented in the study are included in the article/supplementary material, further inquiries can be directed to the corresponding author/s.

## Author Contributions

JC conceived the study, participated in the design and revision, and proofread the manuscript. CW conceived the PLV, PCC, and TE algorithms to extract the brain network connectivity feature and was responsible for EEG feature representation. CM carried out the subject-independent EEG-based emotion classification experiments with the BC-DA-RCNN method, analyzed the experimental results, and drafted the manuscript. ZT carried out the subject-dependent experiments with DA-RCNN and its variants models and analyzed the experimental results. YL carried out the baseline experiments and analyzed the experimental results. XH was responsible for literature review, EEG data preprocessing, and PSD feature extraction. All authors read and approved the final manuscript.

## Conflict of Interest

The authors declare that the research was conducted in the absence of any commercial or financial relationships that could be construed as a potential conflict of interest.

## Publisher’s Note

All claims expressed in this article are solely those of the authors and do not necessarily represent those of their affiliated organizations, or those of the publisher, the editors and the reviewers. Any product that may be evaluated in this article, or claim that may be made by its manufacturer, is not guaranteed or endorsed by the publisher.
